# Supplementary material for: The molecular basis for recognition of 5′-NNNCC-3′ PAM and its methylation state by Acidothermus cellulolyticus Cas9
Source: Nat Commun. 2020 Dec 11;11:6346. doi: 10.1038/s41467-020-20204-1 (PMC7733487; doi:10.1038/s41467-020-20204-1)
Supplement: Supplementary file 1 — Supplementary Information [file 41467_2020_20204_MOESM1_ESM.pdf]

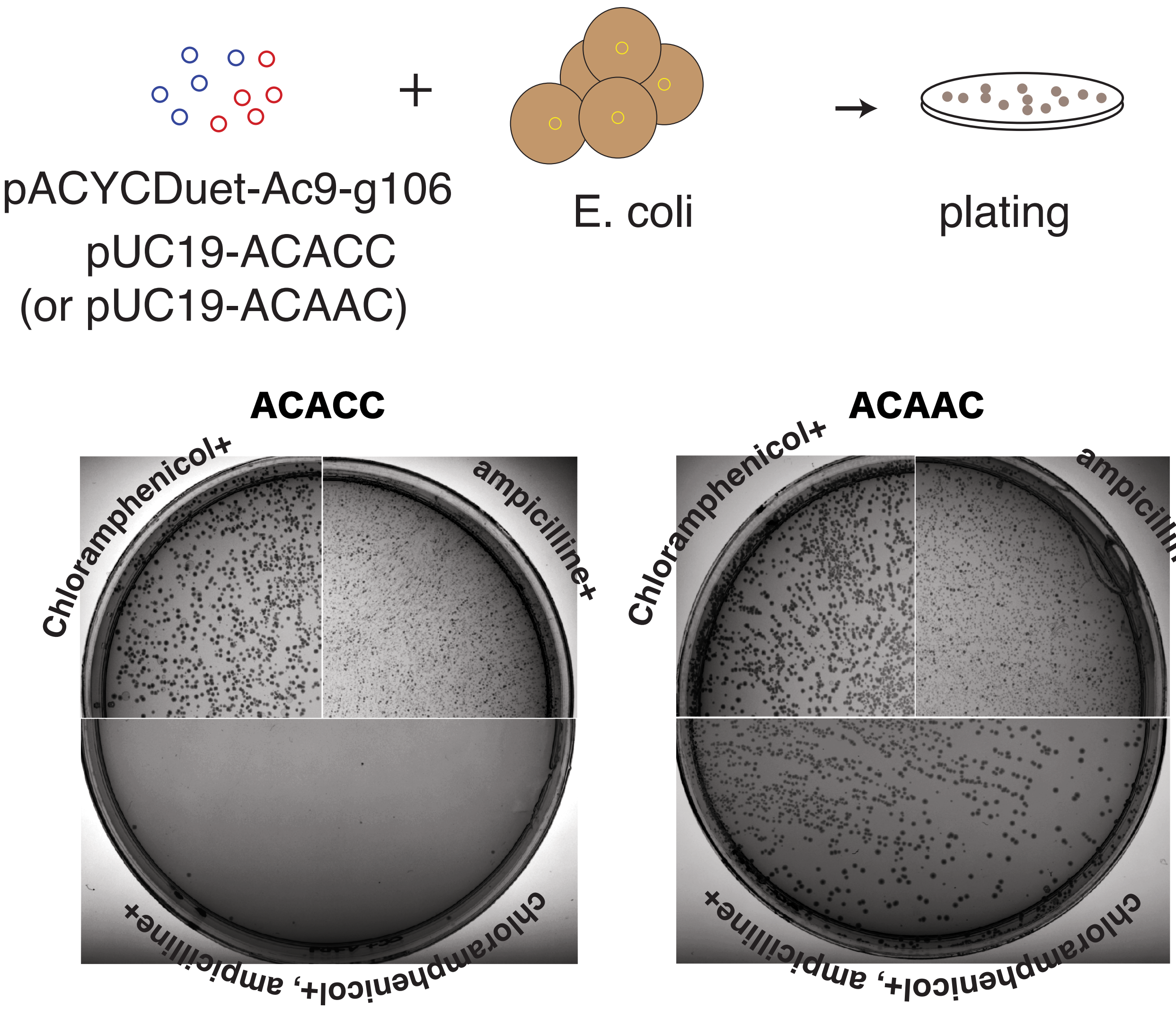

Supplementary Figure 1. The in vivo activity of AceCas9 on plasmids with two different PAMs. The pACYCDuet-Ac9-g106 plasmid (chloramphenicol resistant) encoding both AceCas9 and the single guide RNA sgRNA106 was co-transformed with a pUC19 plasmid (ampicillin resistant) containing the cognate protospacer followed by a PAM sequence (pUC19-ACACC or pUC19-ACAAC) into DH5α competent cells. The cell growth was selected on plates containing either single or double antibiotics.

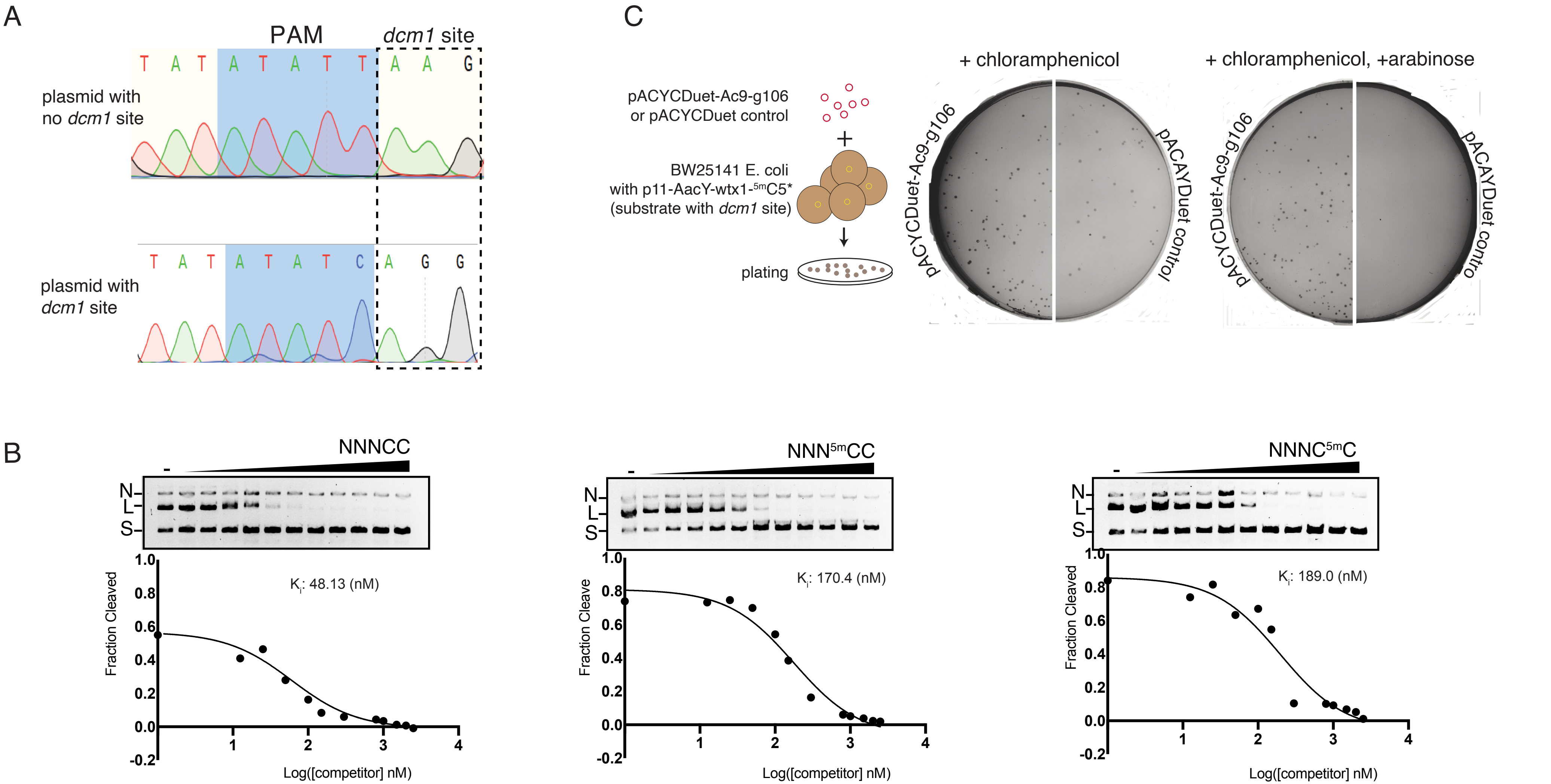

Supplementary Figure 2. The in vivo activity of AceCas9 on the plasmid containing methylation on the 5th nucleotide of the 5'-ACACC-3' PAM, p11-AacY-wtx1-^{5m}C5*. **A.** Confirmation of ^{5m}C by bisulfite sequencing p11-AacY-wtx1-^{5m}C5*. Top, Sequencing read of the PAM followed by non-*dcm1* site, p11-AacY-wtx1. Bottom, Sequencing read of the PAM followed by *dcm1* site, p11-AacY-wtx1-^{5m}C5*. **B.** AceCas9 cleavage competition assay in the presence of three types of double stranded DNA substrate competitors with increasing concentrations: the wild-type (NNNCC), the ^{5m}C4*-containing PAM (NNN^{5m}C4*) or the ^{5m}C5*-containing PAM (NNNC^{5m}C5*). Integrated band intensities from two replicates were used to calculate the fraction of cleavage that was then plotted as a function of competitor concentrations in log scale. Estimated binding constant for each DNA oligo substrate is shown on the graphs. The gel image for one reaction is shown on top, where "N" denotes nicked, "L" denotes linearized, and "S" denotes supercoil DNA. **C.** The ccdB-toxicity-based cell survival assay with p11-AacY-wtx1-^{5m}C5*. The plasmid co-expressing both AceCas9 and its guide RNA, pACYCDuet-Ac9-g106 or a pACYCDuet control plasmid was transformed into BW25141 cells harboring p11-AacY-wtx1-^{5m}C5* followed by plating on either chloramphenicol or chloramphenicol plus arabinose plates.

DNA target — sgRNA — RuvC — HNH — PID — REC

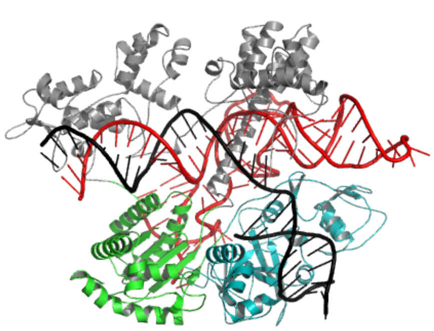

AceCas9, PDBid: 6WBR, this work
charged residues: 28%
PAM: NNNCC

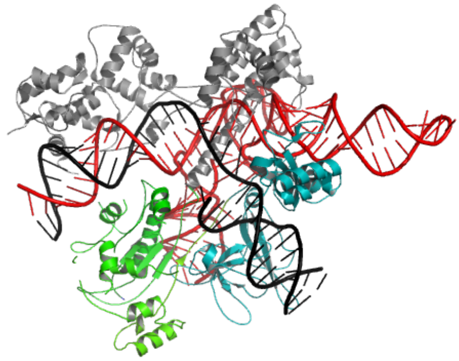

Nme2Cas9, PDBid: 6JE3, Ref. 28
RMSD: 9.4 Å for 650 Ca
sequence identity: 18%
charged residues: 31%
PAM: NNNNCC

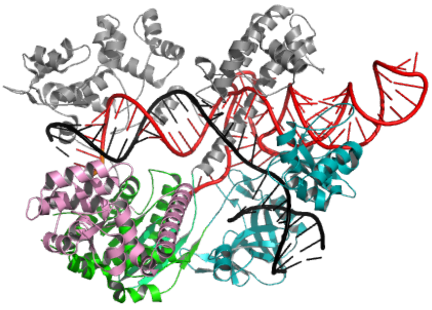

SauCas9, PDBid: 5CZZ, Ref. 23
RMSD: 5.5 Å for 414 Ca
sequence identify: 18%
charged residues: 33%
PAM: NNGRRT

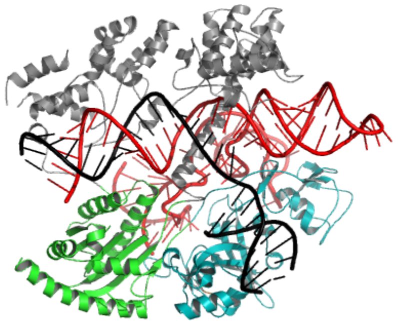

CdiCas9, PDBid: 6JOO, Ref. 27
RMSD: 2.6 Å for 861 Ca
sequence identity: 33%
charged residues: 28%
PAM: NNRHHY

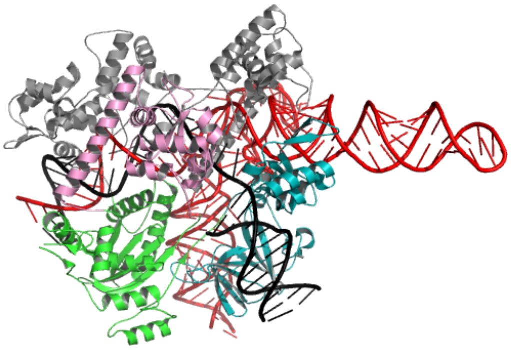

Nme1Cas9, PDBid: 6JDV, Ref. 28
RMSD: 7.1 for 438 Ca
sequence identity: 20%
charged residues: 32%
PAM: NNNGATT

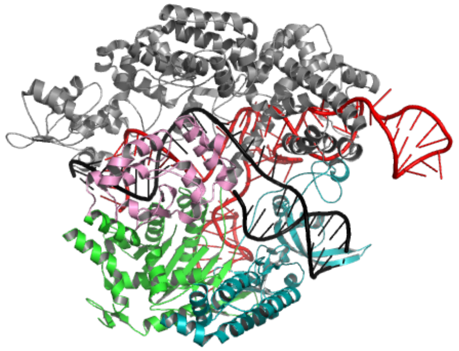

SpyCas9, PDBid: 4UN3, Ref. 22
RMSD: 34.1 Å for 665 Ca
sequence identity: 17%
charged residues: 32%
PAM: NGG

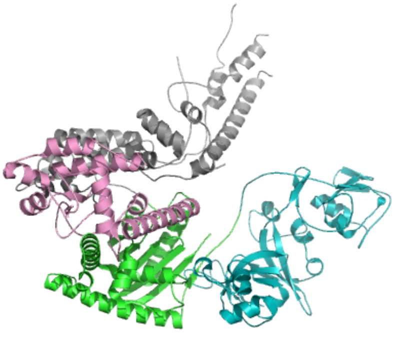

AnaCas9, PDBid: 4OGE, Ref. 24
RMSD: 5.4 Å for 861 Ca
sequence identity: 33%
charged residues: 28%
PAM: unknown

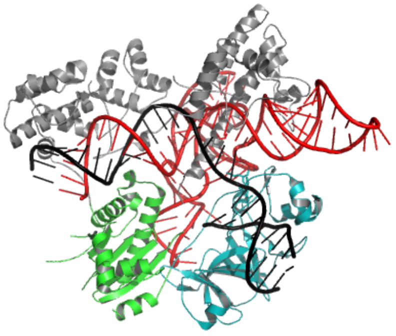

CjeCas9, PDBid: 5X2G, Ref. 26
RMSD: 8.5 Å for 616 Ca
sequence identity: 19%
chargged residues: 34%
PAM: NNNVRYM

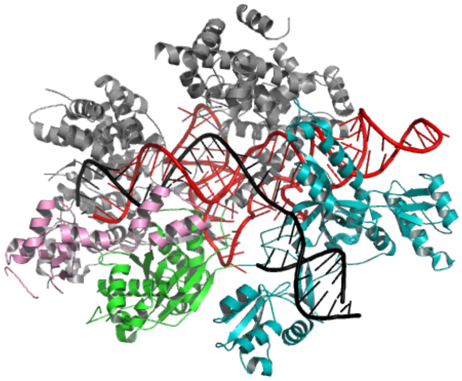

FnoCas9, PDBid: 5B2O, Ref. 25
RMSD: not aligned
sequence identity: 14%
charged residues: 30%
PAM: NGG

Supplementary Figure 3. Structural comparison of currently known Cas9s. Abbreviations used are *Acidothermus cellulolyticus* (Ace), *Streptococcus pyogenes* (Spy), *Neisseria meningitidis* (Nme), *Corynebacterium Diphtheriae* (Cdi), *Campylobacter jejuni* (Cje), *Actinomyces naeslundii* (Ana), *Staphylococcus aureus* (Sau), *Francisella novicida* (Fno). All structures were superimposed (except for FnoCas9) to that of AceCas9 and displayed in the same orientation and colored identically as indicated by the color key on top. FnoCas9 was oriented to a similar orientation. The PDBids, the reference number, RMSD with AceCas9, sequence identity with AceCas9, the percentage of all charged amino acids and the known PAM sequences are denoted directly under each structure.

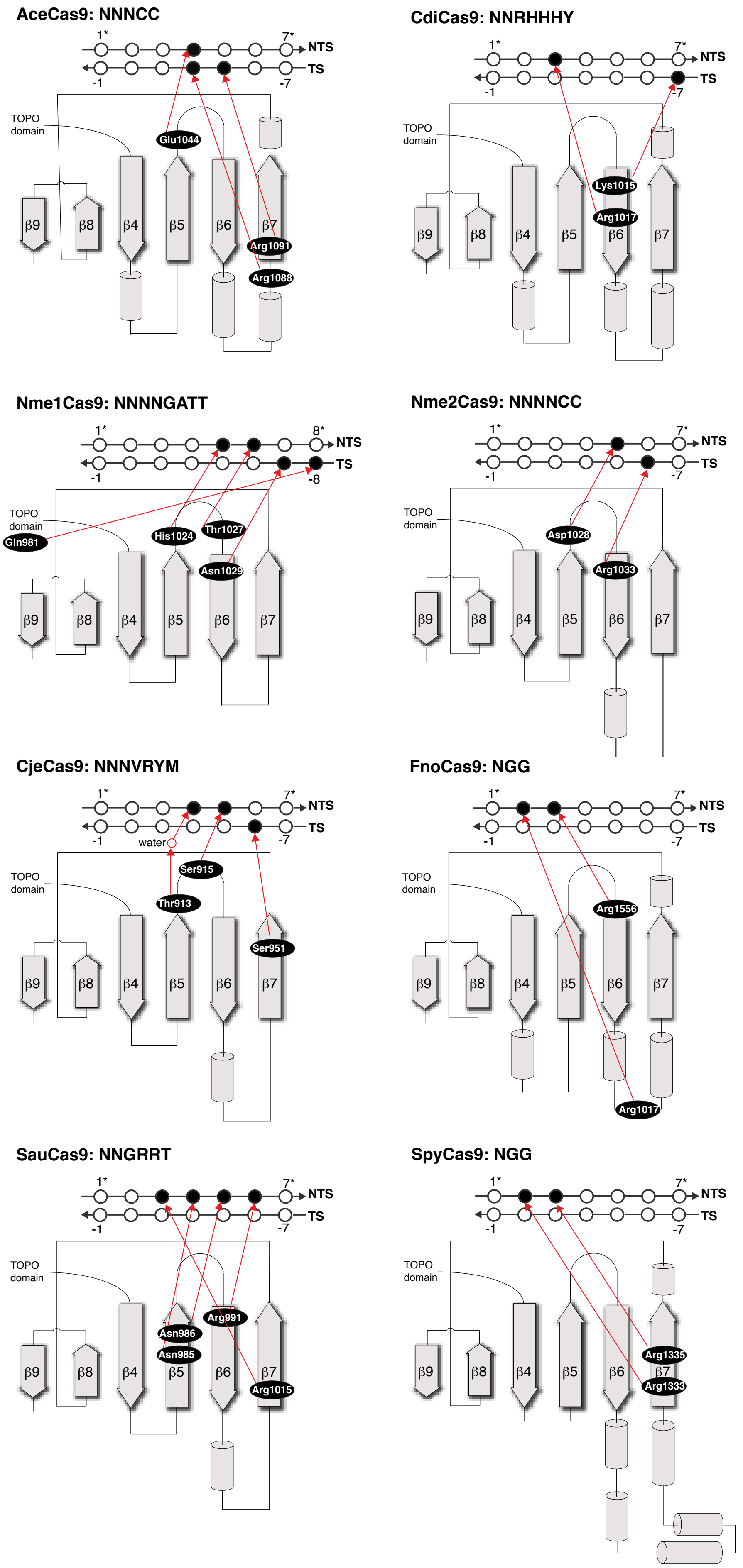

Supplementary Figure 4. Comparison of currently known PAM-Cas9 interactions. The topology of the PAM-interacting subdomain of each Cas9 structure is shown with secondary structure elements labeled. Key residues are shown with respect to the secondary elements and their interactions with PAM nucleotides are indicated by red arrows. NTS, nontarget strand, TS, target strand. For all Cas9s (except for FnoCas9), three important β-strands and their connecting loops comprise of the PAM interacting elements. While the β5 and β7 strand always contact NTS and TS, respectively, β6 can contact both,

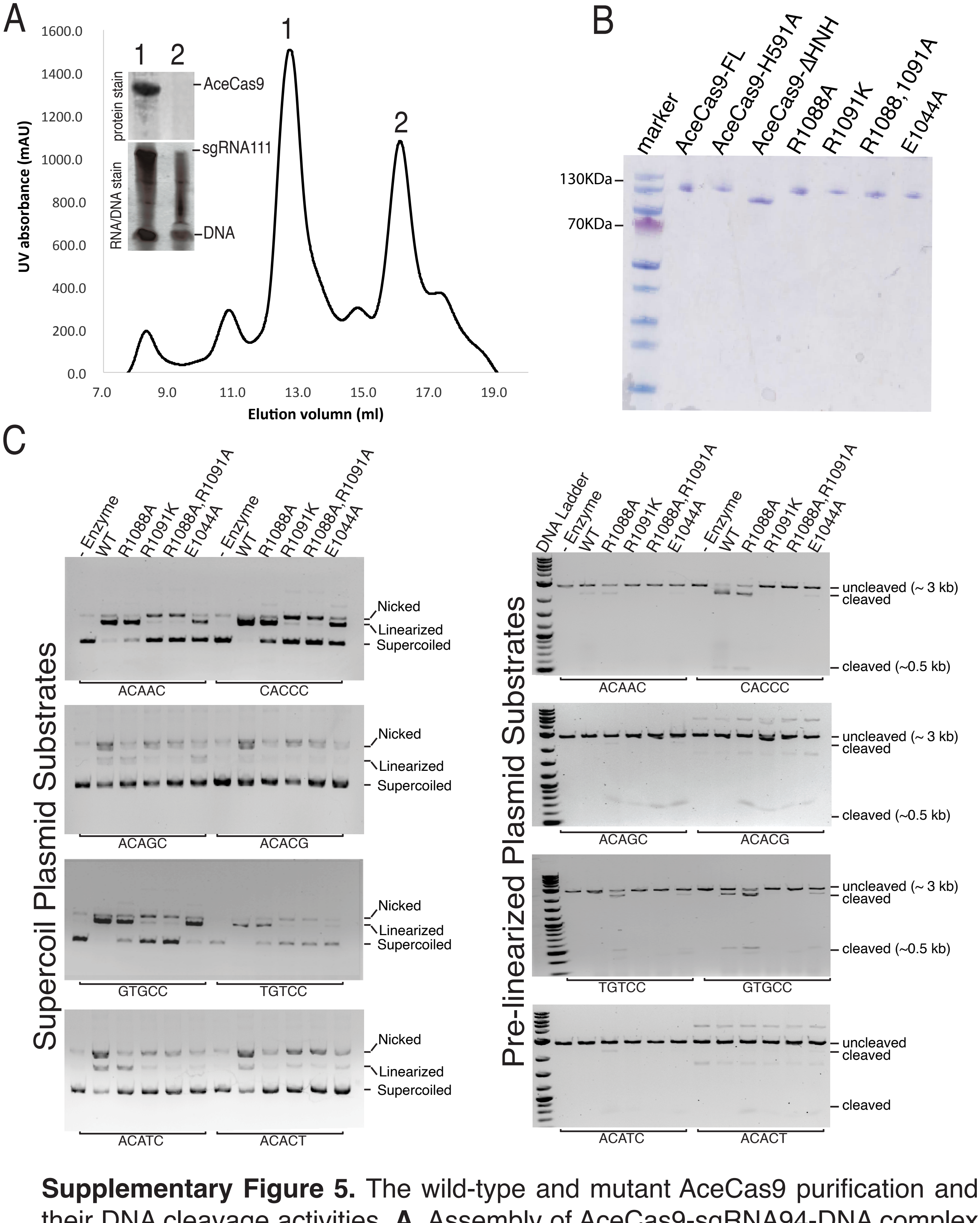

Supplementary Figure 5. The wild-type and mutant AceCas9 purification and their DNA cleavage activities. **A.** Assembly of AceCas9-sgRNA94-DNA complex on a gel filtration column. Fractions containing both the protein and the nucleic acids were collected and pooled. **B.** SDS-PAGE gel evaluation of all AceCas9 used. **C.** In vitro DNA cleavage of the wild-type (WT) and AceCas9 mutants on DNA substrates containing various PAM sequences. Either supercoiled (left) or prelinearized (right) DNA at 6 nM concentration was incubated with 1 μM wild-type or a mutant at 50°C for 15 minutes followed by separation on agarose gel and staining by ethidium bromide. Cropped images were used in Figure 5.

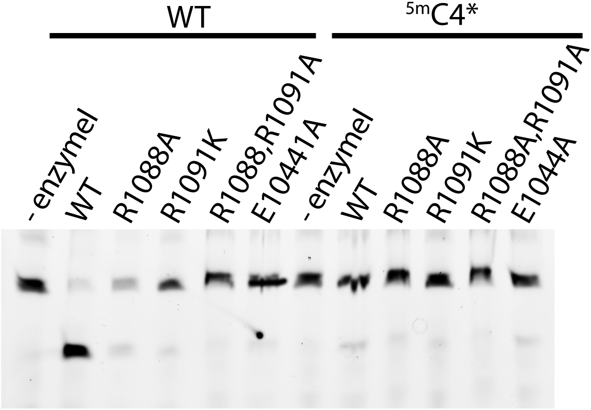

Supplementary Figure 6. Cleavage activities on methylated DNA by the wild-type (WT) or mutant AceCas9. The ^{5m}C4*-containing DNA oligo was annealed with its complementary strand and then incubated with either WT or mutants at 50°C for 1 hours. The cleavage products were separated on a urea PAGE gel and visualized by SYBR Gold staining.

Supplementary Table 1. X-ray Diffraction Data and Refinement Statistics

	AceCas9-ΔHNH-sgRNA-DNA (PDB: 6WBR)	Se-AceCas9-ΔHNH-sgRNA-DNA	AceCas9-ΔHNH-sgRNA-DNA(C4T) (PDB: 6WC0)
**Data collection**			
Space group	$P2_12_12_1$	$P2_12_12_1$	$P2_12_12_1$
Cell dimensions			
a, *b*, *c* (Å)	82.31, 119.35, 177.18	82.97, 119.09, 178.23	81.64, 111.14, 177.62
α, β, γ (°)	90.00, 90.00, 90.00	90.00, 90.00, 90.00	90.00, 90.00, 90.00
Resolution (Å)	2.90 (3.02-2.90)*	3.00 (3.13-3.00)*	3.61 (3.95-3.61)*
R_{merge}	0.178 (2.00)	0.195 (3.38)	0.198 (3.63)
R_{pim}	0.071 (0.781)	0.056 (0.930)	0.058 (1.003)
$I / \sigma I$	12.1 (0.9)	12.4 (0.9)	8.3 (1.0)
CC(1/2)	0.998 (0.362)	1.000 (0.437)	0.997 (0.554)
Completeness (%)	99.7 (97.5)	100.0 (100.0)	99.9 (100.0)
Redundancy	7.2 (7.4)	7.1 (7.3)	7.0 (7.3)
**Refinement**			
Resolution (Å)	2.91 (3.01-2.91)*		3.61 (3.95-3.61)*
No. reflections	38982 (3804)		19328 (4529)
R_{work} / R_{free}	22.4/26.6		23.7/30.4
No. atoms			
Protein	7400		7383
RNA/DNA	2760		2760
Water	0		0
B-factors (Å^2)			
Protein	96.7		183.7
RNA/DNA	113.6		200.8
R.m.s deviations			
Bond lengths (Å)	0.007		0.008
Bond angles (°)	1.110		1.210
Ramachandrian plot (%)			
Favored	93.4		94.1
Outliers	0.0		0.0
Outliers (gly & pro)	0.0		0.4

*Highest resolution shell is shown in parenthesis.

Supplementary Table 2. Frequency Table for All 20 Amino Acids at Four Selected Positions

	**E1044**		**L1075**		**R1088**		**R1091**	
	Library	*Survivor*	*Library*	*Survivor*	*Library*	*Survivor*	*Library*	*Survivor*
**A**	0.08343736	0.21188351	0.00203791	0.03880927	0.13755859	0.02874761	0.10597106	0.031622371
**C**	0	0.00450816	0.02037905	0.01006166	0	0.00143738	0.00203791	0.001437381
**D**	0.81269299	0.36065278	0	0	0	0	0	0
**E**	80.20498	98.3139482	0	0.00862428	0	0	0.00917057	0
**F**	0.1169437	0	0.55329122	0.1696109	0.01426534	0.00143738	0.01018953	0
**G**	0.68983641	0.35163646	0.021398	0.2285435	0.44833911	0.60082506	0.31689423	0.188296848
**H**	0.02890743	0.00450816	0.01426534	0	0.00305686	0.00143738	0.02037905	0
**I**	0	0.00450816	0.02445486	0.00143738	0	0.00143738	0	0
**K**	14.9274029	0.40122622	0.00101895	0	0	0.00143738	0	0
**L**	0.00262795	0.00450816	96.5549215	21.8050625	1.18096597	1.74354257	0.95577746	0.097741875
**M**	0.0965771	0	0.93743632	0.06180736	0.00101895	0	0.00203791	0
**N**	0.09986203	0.00450816	0	0	0	0	0	0
**P**	0.00459891	0.00901632	0.01324638	0.00287476	0.17424088	0.06899426	0.06419401	0.068994265
**Q**	0.43229748	0.04057344	0.01324638	0	0.93234155	0.04024665	0.94762584	0.024435469
**R**	1.67334604	0.21188351	0.01324638	0.02299809	96.180966	97.4127151	95.8396169	99.40779923
**S**	0.01051179	0	1.03117995	0.72012764	0.00407581	0.00574952	0.00407581	0.004312142
**T**	0.08803627	0.00901632	0.00509476	0	0	0	0.00305686	0.002874761
**V**	0.69180737	0.06311424	0.51355207	76.8495494	0	0.03593451	0.00407581	0.001437381
**W**	0.02890743	0.00450816	0.27715508	0.08049331	0.92317098	0.05605784	1.71489709	0.171048282
**Y**	0.00722686	0	0.00407581	0	0	0	0	0

Supplementary Table 3. DNA or RNA Oligoes Used

Description	Sequence (5'to 3')	Used for
**T7 Promoter**	TAATACGACTCACTATAGG	In vitro transcription of guide RNAs, non-template strand
**Single guide RNA 123 template**	TAATACGACTCACTATAATCCCTAGCGTAATGCTAGA<br>TTATGGGATGCTGGGGAGCCTGAAAAGGCTACCTAGC<br>AAGACCCCTTCGTGGGGTCGCATTCTTCACCCCCTCGC<br>AGCAGCGAGGGGGTTC	In vitro transcription of guide RNA, template strand. RNA is used for cleavage assays (Fig. 1, 2, 3, 4, 5)
**Single guide RNA 111 template**	TAATACGACTCACTATAGGTAGGATGGCAAGATCCTG<br>GTATGCTGGGGAGCCTGAAAAGGCTACCTAGCAAGAC<br>CCCTTCGTGGGGTCGCATTCTTCACCCCCTCGCAGCAG<br>CGAGGGGGTTC	In vitro transcription of guide RNA, template strand. RNA is used for cleavage assays and co-crystallization (Fig. 2, 3, 6)
**Target strand-1 ATACC PAM**	CGCCAGGTATATACCAGGATCTTGCCATCC	Co-crystallization<br>(Fig. 2, 3, 6)
**Non target strand-1 ATACC PAM**	ATACCTGGCG	Co-crystallization<br>(Fig. 2, 3, 6)
**Target strand-2 ATATC PAM**	CGCCAGATATATACCAGGATCTTGCCATCC	Co-crystallization<br>(Fig. 6)
**Non target strand-2 ATATC PAM**	ATATCTGGCG	Co-crystallization<br>(Fig. 6)
**Target strand-3 ACACC PAM**<br>**Non target strand-3 ACACC PAM**	GGTAGGATGGCAAGATCCTGGTATACACC<br>GGTGTATACCAGGATCTTGCCATCCTACC	In vitro cleavage assay<br>(Fig. 1B, 2, 4C, 5, S2B)
**Target strand-4 ACAAC PAM**<br>**Non target strand-4 ACAAC PAM**	GGTAGGATGGCAAGATCCTGGTATACAAC<br>GTTGTATACCAGGATCTTGCCATCCTACC	In vitro cleavage assay<br>(Fig. 1B, 5, S2B)
**Target strand-5 CACCC PAM**<br>**Non target strand-5 CACCC PAM**	GGTAGGATGGCAAGATCCTGGTATCACCC<br>GGGTGATACCAGGATCTTGCCATCCTACC	Invitro cleavage assay<br>(Fig. 1B, 5, S2B)
**Target strand-6 GTGCC PAM**<br>**Non target strand-6 GTGCC PAM**	GGTAGGATGGCAAGATCCTGGTATGTGCC<br>GGCACATACCAGGATCTTGCCATCCTACC	Invitro cleavage assay<br>(Fig. 5, S2B)
**Target strand-7 TGTCC PAM**<br>**Non target strand-7 TGTCC PAM**	GGTAGGATGGCAAGATCCTGGTATTGTCC<br>GGACAATACCAGGATCTTGCCATCCTACC	Invitro cleavage assay<br>(Fig. 5, S2B)
**Target strand-8 ACACG PAM**<br>**Non target strand-8 ACACG PAM**	GGTAGGATGGCAAGATCCTGGTATACACG<br>CGTGTATACCAGGATCTTGCCATCCTACC	Invitro cleavage assay<br>(Fig. 5, S2B)
**Target strand-9 ACAGC PAM**<br>**Non target strand-9 ACAGC PAM**	GGTAGGATGGCAAGATCCTGGTATACAGC<br>GCTGTATACCAGGATCTTGCCATCCTACC	Invitro cleavage assay<br>(Fig. 5, S2B)
**Target strand-10 ACACT PAM**<br>**Non target strand-10 ACACT PAM**	GGTAGGATGGCAAGATCCTGGTATACACT<br>AGTGTATACCAGGATCTTGCCATCCTACC	Invitro cleavage assay<br>(Fig. 5, S2B)
**Target strand-11 ACATC PAM**<br>**Non target strand-11 ACATC PAM**	GGTAGGATGGCAAGATCCTGGTATACATC<br>GATGTATACCAGGATCTTGCCATCCTACC	Invitro cleavage assay<br>(Fig. 5, S2B)
**Target strand-12 ACATC PAM**<br>**Non target strand-12 ACATC PAM**	GGAGGCAAGATCCTGGTATCCACCTTAGC<br>GCTAAGGTGGATACCAGGATCTTGCCTCC	In vitro cleavage assay<br>(Fig. 1C, S3)
**Target strand-13 ACA^{5m}CC PAM**<br>**Non target strand-13 ACA^{5m}CC PAM**	GGAGGCAAGATCCTGGTATCCA^{5m}CCTTAGC<br>GCTAAGGTGGATACCAGGATCTTGCCTCC	In vitro cleavage assay<br>(Fig. 1C, S3)
**Target strand-14 ACAC^{5m}C PAM**	GGAGGCAAGATCCTGGTATCCAC^{5m}CTTAGC	In vitro cleavage assay<br>(Fig. 1C)
**Target strand-15 ACA^{5m} C^{5m}C PAM**	GGAGGCAAGATCCTGGTATCCA^{5m}C^{5m}CTTAGC	In vitro cleavage assay<br>(Fig. 1C)
**Target strand-16 ACA^{5m}CC PAM**	GGTAGGATGGCAAGATCCTGGTATAGG^{5m}CC	In vitro cleavage assay<br>(Fig. 1D)

**Ac9dHNH**	AGCGCGGGCGGTTACGCAGCTGTCGCGCTAAG	Q5 primer for AceCas9ΔHNH clone
**Ac9h591_F**<br>**Ac9h591_R**	GAATTCGGAGCTTGACGCGATTGTCCCGCGTACG<br>CGTACGCGGGACAATCGCGTCAAGCTCCGAATTC	Forward (F) and Reverse (R) Q5 primers for H591A mutant
**Ac9E1044_F**<br>**Ac9E1044_R**	GGCTTTGCGGATGATAAAAGGATCAATCTTAAGCCG<br>TGTGACGACCCAATGCCGCTC	F and R primers to make E1044A mutant
**Ac9R1088A_F**<br>**Ac9R1088A_R**	CAATTCTTCCCGGCGTGTTGGCGGGCGAC<br>TGCGAGAATCTCCCCGGCTTC	F and R primers for R1088A mutant
**Ac9R1091A_F**<br>**Ac9R1091A_R**	GGTGTTGGGCGGCGACCGTCGCAAAGG<br>GCGGGAAGAATTGTGCGAGAATC	F and R primers for R1091A mutant
**Ac9R1088K_F**<br>**Ac9R1088K_R**	TTCTTCCCGAAGTGTTGGCGG<br>TTGTGCGAGAATCTCCCCG	F and R primers for R1088K mutant
**Ac9R1091K_F**<br>**Ac9R1091K_R**	TGTTGGAAGGCGACCGTC<br>CCGCGGGAAGAATTGTGC	F and R primers for R1091K mutant
**fPID_F**<br><br>**fPID_R**	TCGTCGGCAGCGTCAGATGTGTATAAGAGACAGCCG GGGATTTAAGTATGTTCCTG<br>GTCTCGTGGGCTCGGAGATGTGTATAAGAGACAGGA CCAAGCGCAGTTCGTCG	F and R primers for focused PID library
**ccDBAC_F**<br>**ccDBAC_R**	GCAAGATCCTGGTATACAACAAGCTTGG<br>CATCCTACCTCTAGAGCGTGATATTACCCTG	F and R primers for ACAAC ccDB clone
**Sglib_F**<br>**Sglib_R**	CACTATAGGGGTAGGATGGCAAGATCCTGGTAT<br>CCAAGGGGTTATGCTAGTTATTGCTCAGCGG	F and R primers for guide RNA library
**PAMlib_F**<br>**PAMlib_R**	GGTAAGAAACCATTATTATCATGACATTAACC<br>NNNNNNATACCAGGATCTTGCCATCCTACCGACGTC AGGTGGCACTTTTC	F and R primers for PAM library
**GG_F**<br>**GG_R**	CCTGGTATAGGCCAAGCTTGGCTG<br>ATCTTGCCATCCTACCTCTAGAGCGTG	F and R primers for AGGCC PAM clone
**TGT_F**	GAT CCT GGT ATT GTC CAT TAA AAAACC	F Primer for TGTCC PAM
**CAC_F**	GAT CCT GGT ATC ACC CAT TAA AAAACC	F Primer for CACCC PAM
**GTG_F**	GAT CCT GGT ATG TGC CAT TAA AAAACC	F Primer for GTGCC PAM
**NNN_R**	TTG CCA TCC TAC CGA CGT CAG	R Primer for TGTCC, CACCC, GTGCC PAM
**pUCAC_F**<br>**pUCAC_R**	GAT CCT GGT ATA CAA CAT TAA AAAACC A<br>TTG CCA TCC TAC CGA CGT CAG GTG G	F and R Primers for ACAAC PAM in pUC19
