# Supplementary material for: The molecular basis for recognition of 5′-NNNCC-3′ PAM and its methylation state by Acidothermus cellulolyticus Cas9
Source: Nat Commun. 2020 Dec 11;11:6346. doi: 10.1038/s41467-020-20204-1 (PMC7733487; doi:10.1038/s41467-020-20204-1)
Supplement: Supplementary file 6 — Description of Additional Supplementary Files [file 41467_2020_20204_MOESM6_ESM.docx]

**Legends for Supplementary Data files**

**Supplementary Data File 1.** PAM depletion scores for all possible 2mer nucleotide combinations

**Supplementary Data File 2.** Computed frequencies of non wild-type nucleotides at each position of the input (lib) and the survivor (srv) RNA libraries.

**Supplementary Data File 3.** Computed Percent Difference Frequency for Each of the 20 Amino Acids at Each Residue Position
