# Supplementary material for: The molecular basis for recognition of 5′-NNNCC-3′ PAM and its methylation state by Acidothermus cellulolyticus Cas9
Source: Nat Commun. 2020 Dec 11;11:6346. doi: 10.1038/s41467-020-20204-1 (PMC7733487; doi:10.1038/s41467-020-20204-1)

**Source Data Files**

**The Molecular Basis for Recognition of 5'-NNNCC-3' PAM and Its Methylation State by *Acidothermus cellulolyticus* Cas9**

Anuska Das^1^, Travis H. Hand^1^, Chardasia L. Smith^1^, Ethan Wickline^2^, Michael Zawrotny^1^, and Hong Li^1,2^*

^1^Institute of Molecular Biophysics, ^2^Department of Chemistry and Biochemistry, Florida State University, Tallahassee, FL 32306, USA.

*corresponding author: [hong.li@fsu.edu](mailto:hong.li@fsu.edu)

Panel 1. A plasmid DNA (pUC19) cleavage gel image with DNA marker ( 1 kb DNA ladder, Sigma) similar to those shown in Figure 1B, top, Figure 2A, Figure S2B, and Figure S5C, left. Ethidium bromide was used for staining and visualized by Bio-Rad ChemiDoc Imaging system.

Panel 2. Original gel images for those used in Figure 1B. Ethidium bromide was used for staining and visualized by Bio-Rad ChemiDoc Imaging system.

Supercoiled DNA gel


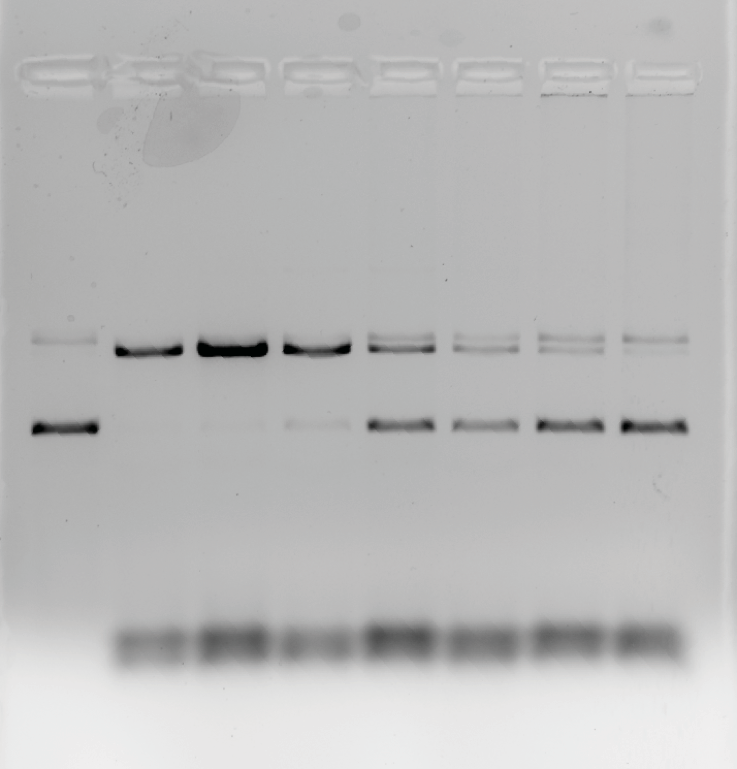


Linearized (L)

Supercoiled (S)

Prelinearized DNA gel


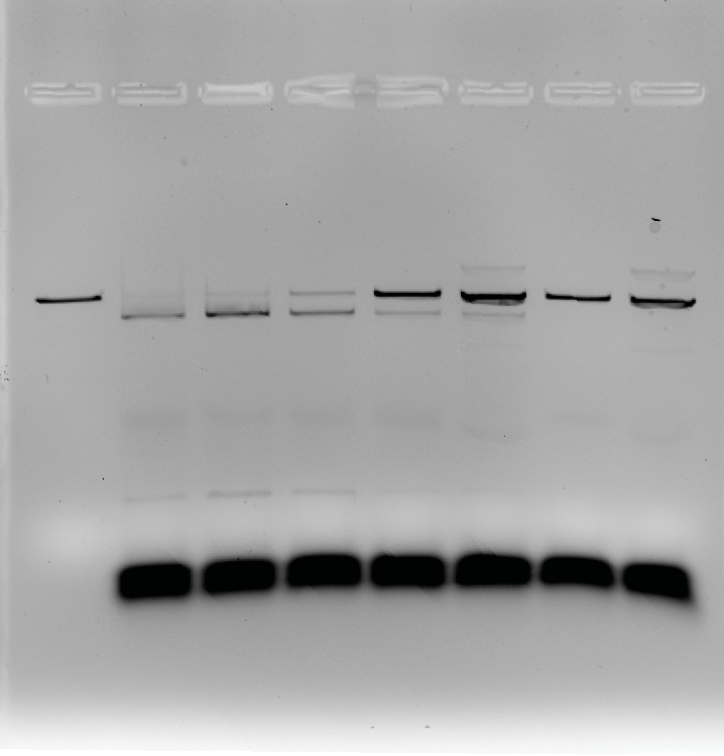


Uncleaved

Cleaved

Panel 3. Original images used for Figure 1C and Figure 1D. Top, the target DNA strand is not methylated and contains a 5´-hexachlorofluorescein (HEX) tag for visualization; Bottom, ethidium bromide was used for staining and visualized by Bio-Rad ChemiDoc Imaging system.


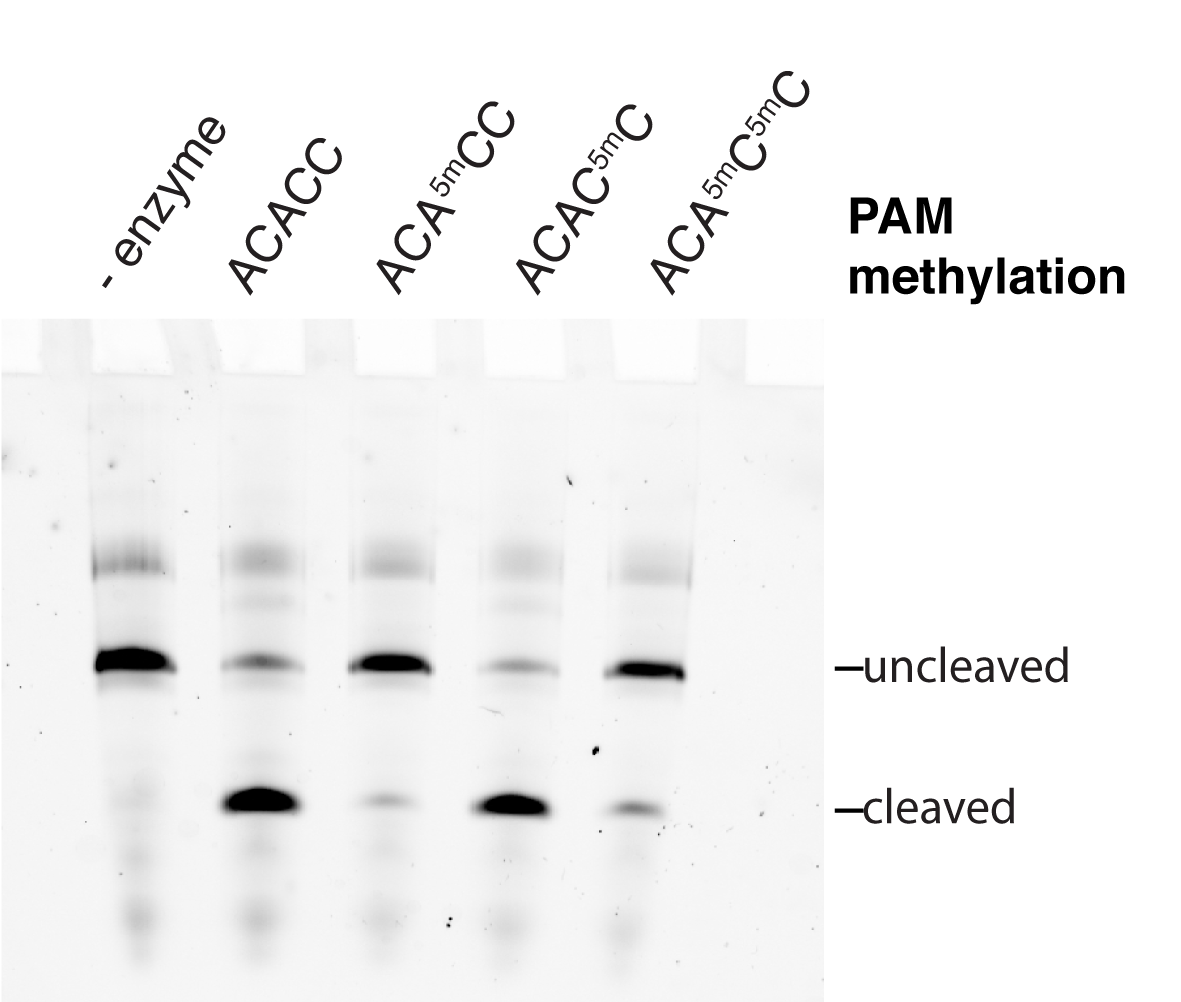


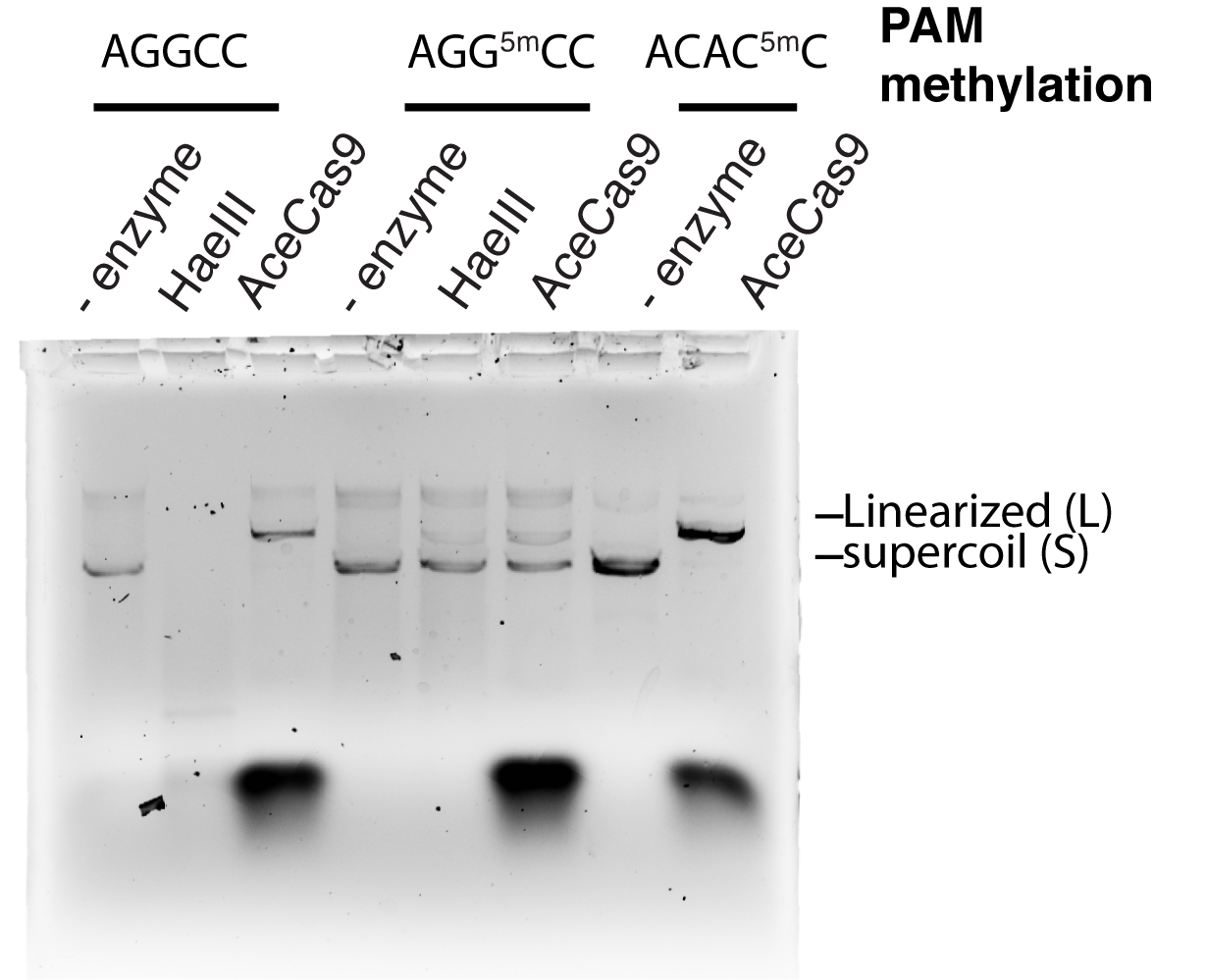


Panel 4. Original gel image used for Figure 2A. Ethidium bromide was used for staining and visualized by Bio-Rad ChemiDoc Imaging system.


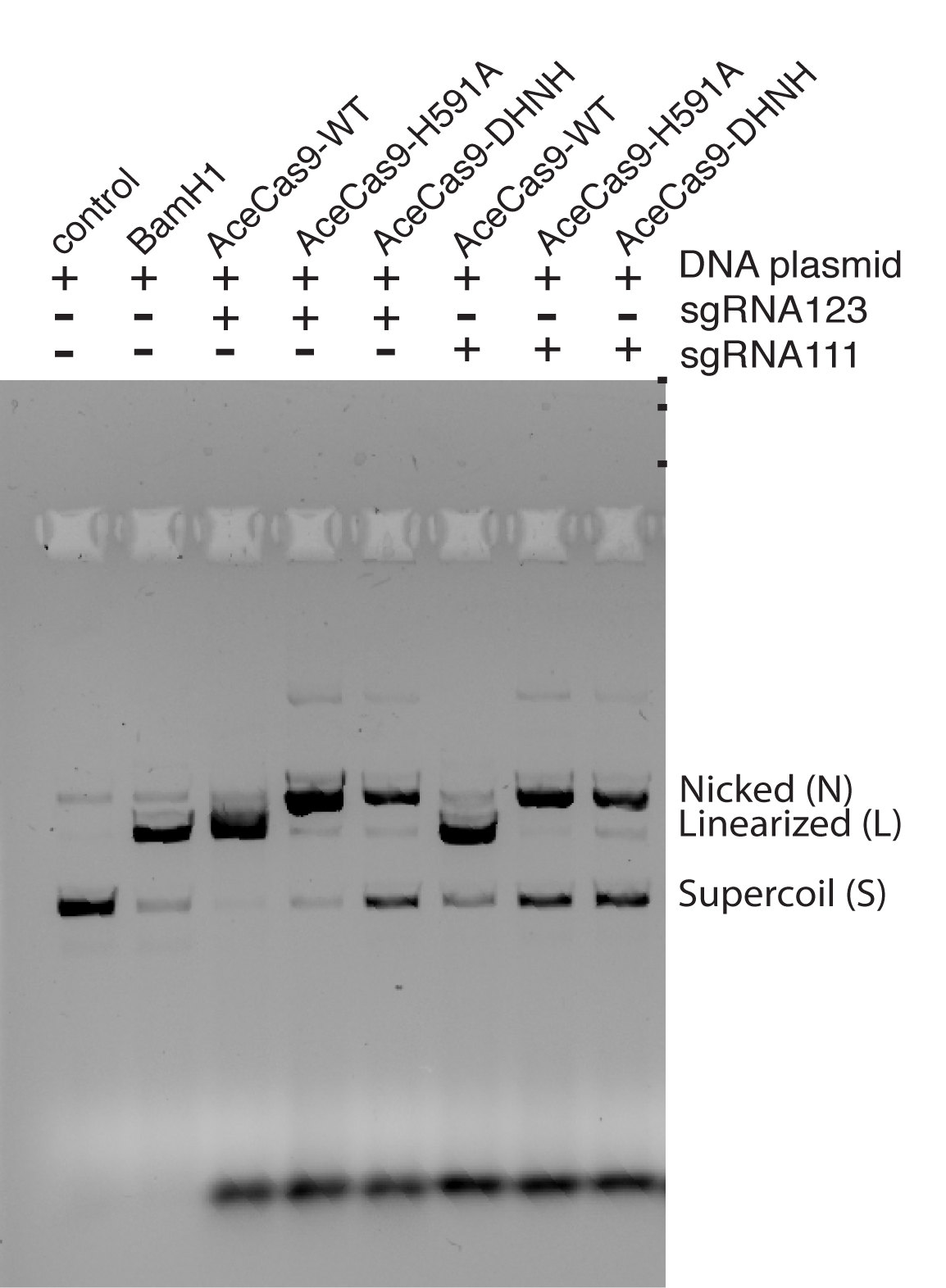


Panel 5. Original gel image used for Figure 4B. Ethidium bromide was used for staining and visualized by Bio-Rad ChemiDoc Imaging system.


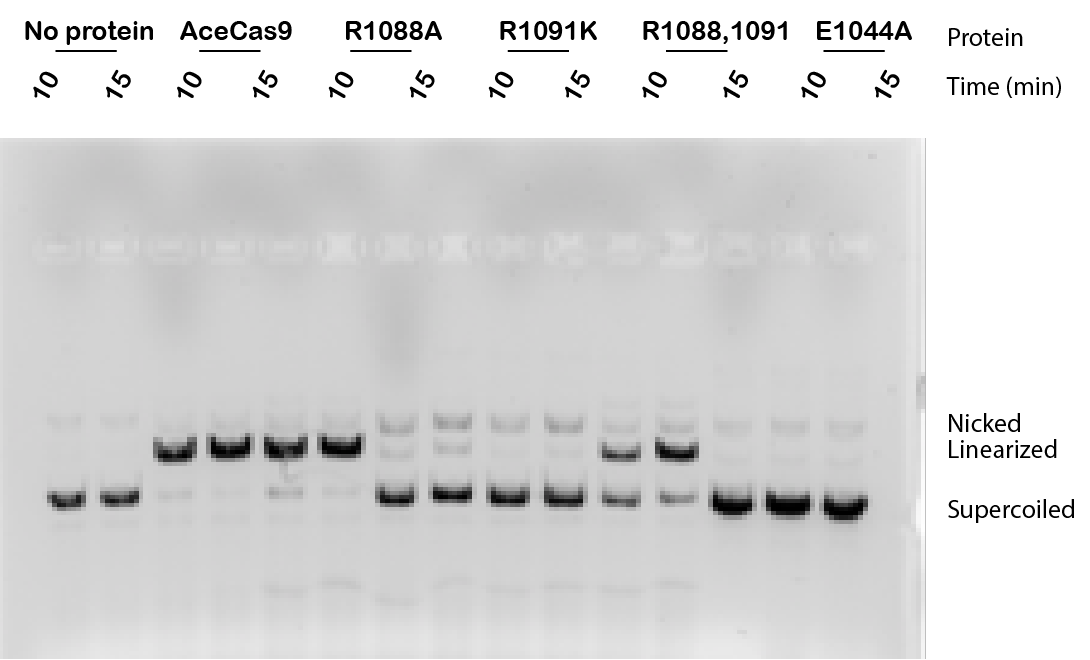

Supplement: Supplementary file 7 — Source Data [file 41467_2020_20204_MOESM7_ESM.docx]
